# Effects of incorporating oasis by-products on fattening performance and carcass characteristics of Ouled Djellal lamb

**DOI:** 10.14202/vetworld.2018.1397-1403

**Published:** 2018-10-09

**Authors:** Abdelhamid Baa, Fodil Arbouche, Rafik Arbouche, Etienne Montaigne, Yasmine Arbouche, Halima Saâdia Arbouche

**Affiliations:** 1 Department of Agronomy, Faculty of Science of Nature and Life, University of El Tarf, El Tarf, Algeria; 2 Department of Agronomy, Faculty of Science of Nature and Life, University of M’sila, M’Sila, Algeria; 3 Department of Agronomy, Faculty of Science of Nature and Life, University of Ghardaia, Ghardaia, Algeria; 4 Joint Research Unit “Market, Organization, Institution”, Actors Strategies, University Supagro of Montpellier, Montpellier, France; 5 Department of Agronomy, Faculty of Science of Nature and Life, University of Sétif, Sétif, Algeria

**Keywords:** cull dates, date pedicels, extract of rumen content, sheep fattening

## Abstract

**Aim:**

The aim of this study was to determine the effects of incorporating three local oasis by-products [cull dates (CDs), date pedicels (DPs) treated with urea, and juice from rumen content] into the food ration of Ouled Djellal lambs on fattening performance and carcass characteristics.

**Materials and Methods:**

The experiment was carried out over 105 days, with an adaptation period of 15 days, on four groups each consisting of 10 male Ouled Djellal lambs aged 7-8 months with an average live body weight of 32±1.5 kg, randomly distributed, and raised in tie stalls. The basic rations were formulated at a rate of 0% (control), 50%, 80%, and 100% substitution of barley straw by DPs treated with urea. The additional rations intended for the experimental groups consisted of 100% substitution of corn by CDs. Before distributed, they were sprayed with rumen content extract (RCE) at a rate of 250 ml/kg.

**Results:**

The 100% group displayed a highly significant difference compared to the other groups, with a live body weight of 43 kg (p<0.05), an average daily gain of 191 g, and feed efficiency of 5.08. These three parameters developed in proportion to the rates of incorporation of CDs and pedicels treated with urea. The carcass yield of the 100% group (48.7%) is significantly higher than the other groups while the thickness of back fat is significantly lower. Economically speaking, the profit margin of the 100% group is 30.93 Algerian dinars (DZD) per day per animal.

**Conclusion:**

The use of by-products of the date palm (CDs and pedicels) combined with RCE in animal feed with a view to fattening sheep, in particular in oasis zones, represents an alternative in enhancing growth performances and carcass characteristics and offers a relatively cheap prospect for the availability of red meat for populations with low purchasing power.

## Introduction

Reducing the cost price of 1 kg of ovine meet requires a reduction in the cost of feed, which accounts for >47% of the overall production cost [1]. In light of the global economic crisis and the fall in oil prices, emerging countries such as Algeria, which imported all the raw materials used for livestock feed formulas, are forced to find solutions capable of reducing imports while maintaining the purchasing power of the poorest sections of the population. The use of non-conventional foods, such as agricultural and agro-industrial by-products, is a possible solution, even if improving their nutritional qualities requires chemical treatment [2,3]. In oasis zones, the volume of products discarded by packing facilities and of cull from the date processing units (date paste) is quite considerable [4].

Algeria has large quantities of agro-industrial by-products which can be recycled as animal feed, in particular, the by-products of apricot processing in semiarid zones [5] and the by-products of date palms in oasis zones [6,7] comprising pedicels, palms, cull dates (CDs), and pits which can form the rations of small ruminants [8,9].

In the abattoirs, the rumen content is tipped into landfill sites and represents a source of pollution estimated at an average of 25 kg per slaughtered animal [10]. Recycling this content could be feasible as its chemical composition totals, on average, 13.4 kg of dry matter (DM), 21.8% crude protein (of which 73.4% amino acids), 30.3% raw fiber, 6.1% fat, and 11.5% ash [11].

Rumen content has been used in animal feed [12,13]. Extracts of rumen content improve livestock farming performances [14].

The present study examined the possibility of incorporating three local oasis by-products: Date pedicels (DPs) treated with urea substituted for barley straw in the basic ration and CDs substituted for corn in the additional ration, with the latter soaked in the rumen content extract (RCE) when distributed.

## Materials and Methods

### Ethical approval

The present study was conducted after approval of the Institutional Animal Ethics Committee Laboratory of the Agriculture Department, Ghardaia University, Algeria.

### Diets, animals, and experimental protocol

The three oasis by-products used in the experiment come from the region of Sidi Okba (Wilaya of Biskra). CDs of the “Deglet Nour” variety and DPs from a whole year were collected from the packing facilities in the region of Sidi Okba (Wilaya of Biskra in South-East Algeria). The CDs were crushed using a hammer mill fitted with a 1 mm grate. The chemical composition is presented in Table-1.

**Table-1:** Chemical composition of CDs.

Mineral substances (% of DM)	2.9
Total nitrogenous matter (% of DM)	4.2
Crude fiber (% of DM)	9.4
Fat (% of DM)	8.2
Total sugars (% of DM)	63.87
Gross energy (kcal/kg of DM)	4.235
Meat feed unit	1.12

CDs=cull dates DM=Dry matter

The DPs from 1 year were chopped and made into bundles of 10-15 kg to facilitate treatment with urea (Table-2) [9].

**Table-2:** Chemical composition of DPs[9].

Designation	DM %	% of DM			

		OM	TNM	CF	MM
Pedicels from 1 year treated under black plastic film	86.28	94.88	13.62	32.18	5.12

DPs=Date pedicels. DM=Dry matter, OM=Organic matter, TNM=Total nitrogenous matter, CF=Crude fiber, MM=Ash

Containing 46.6% nitrogen, urea was used for the chemical treatment process. The solution contained 70 g urea/L of water for 1 kg DM [6]. The treatment lasted 35 days in a dark, anaerobic environment created using a black plastic film at a temperature of between 34°C and 37°C.

The rumen content was collected from the sheep abattoirs immediately after the animals were gutted. After being spread on the plastic film, the rumen content was treated with a 1% solution of hydrochloric acid to prevent flies from laying any eggs. It was dried in the fresh air after the rumen matter had been turned several times. The RCE was obtained by adding 1 L of water heated to 80°C/kg of rumen content (L/kg) and kneading every 6 h for 24 h. After filtering, the extract was placed in a fridge at a temperature of 6°C. The nitrogenous chemical composition was determined according to the KJELDAHL method and the amino acids by high-performance liquid chromatography. The results are shown in Table-3.

**Table-3:** Content of the ovine RCE in terms of nitrogenous matter (% of DM) and amino acids (% of TNM).

RCE (%)	TNM	Amino acid content								

		Cystine	Methionine	Threonine	Lysine	Glutamic acid	Aspartic acid	Arginine	Leucine	Isoleucine
	8.32	0.89	1.10	2.14	2.56	10.21	6.24	2.85	4.36	1.81

RCE=Rumen content extract, DM=Dry matter, TNM=Total nitrogenous matter

The test was carried out on the pilot farm of Yahïa Ben Aïchouche (Algeria) where 40 Ouled Djellal lambs aged 7-8 months and weighing 32 ± 1.5 kg were randomly divided into four groups of 10 according to diet and housed in tie stalls. All the animals were dewormed and identified by means of tags before the experiment was launched. The test lasted for 105 days, with a 15-day adaptation period.

The basic ration comprised barley straw, oat hay, and treated DPs (Table-4). In the experimental groups, the barley straw was substituted by treated pedicels at a rate of 50% (group 50), 80% (group 80), and 100% (group 100). The basic ration was distributed as follows:

**Table-4:** Composition and nutrient content of the basic ration.

Ingredients (g)	Control	Group 50	Group 80	Group 100
Barley straw	700	350	140	0
Treated pedicels		350	560	700
Oat hay	700	700	700	700
Nutrient content as %				
Dry matter	94.75	92.75	91.55	90.75
Fat	1.5	1.5	1.5	1.5
Crude protein	3.95	6.75	8.43	9.55
Crude fiber	43.9	41.425	39.94	38.95
Mineral substances	5.45	5.45	5.45	5.45
MFU	0.22	0.24	0.25	0.26

MFU=Meat feed unit

- Morning: Barley straw and treated DPs
- Evening: Oat hay.

Two types of additional ration were prepared (Table-5) [15], with the control group receiving a commercial ration made from corn (60%) and the experimental groups receiving a ration, in which the corn was entirely replaced by CDs. The additional ration of the experimental groups was sprayed with 250 ml of filtrate/kg and distributed immediately to offset the deficit in nitrogenous matter in the CDs.

**Table-5:** Feed formulae of the additional rations (in % 100 of feed).

Ingredients	Control group	Experimental groups
Corn	60	0
Cull dates	0	60
Soya meal	20	20
Wheat bran	9	9
Carob	9	9
Salt	1	1
CMV	1	1
Rumen fluid (ml/kg)	0	250
Nutrient content as %		
Dry matter	85.31	87.71
Fat	2.59	2.61
Crude protein	16.23	16.25
Crude fiber	7.53	7.90
Mineral substances	6.83	6.34
MFU*	1.14	1.15

* Calculated according to the equations proposed by Sauvant *et al*. [15], CMV=Complement mineral vitamin, MFU=Meat feed unit

The animals were weighed individually every month at a specific time after 12 h without food using an electronic weight crate with a maximum weight of 100 kg and precise to 5 g.

The basic ration was distributed *ad libitum*, limiting refusal to between 5% and 10%. The concentrate was dispensed at a rate of 2 kg/100 kg of live weight (LW) and readjusted by 5% at the end of the test by maintaining a refusal of 100 g/animal/day. A mineral lick was made freely available to each group at watering.

At the end of the test, all the animals in each group were slaughtered after a period of 12 h without food. The carcasses were weighed hot (HC) and then placed in a cold room at 4°C to determine the cold carcass weight (CC). The thickness of back fat was also measured on the CC [16]. Echoing, Boccard and Dumont [17], other measurements were taken, indicating the width of the pelvis (G), width of the thorax (LAC), width of the shoulders (M), depth of the breast (TH), length of the carcass (K), length of the leg (F), and thickness of the leg. The density index for the leg (width/length) and carcass (width/length), the muscle index (thickness over length of leg), the carcass index (weight over length of carcass), and the conformation index (g/cm), which is the sum of the two previous indices, were also calculated.

### Statistical analysis

The descriptive statistics and the variance analysis of the univariate general linear model (ANOVA) were implemented using the Statistical Package for the Social Sciences software (SPSS version 21, IBM, USA), to analyze the LW, the average daily gain (ADG), the average daily intake (ADI), and the feed conversion ratio (FCR). The general linear model was used to test the effects of the factors (substitutions) on the variables, while the *post hoc* test was used by applying the Student–Newman–Keuls and Duncan tests to estimate the significance or homogeneity between the different subgroups (comparison test between averages). The differences are deemed to be significant with an error risk of 5%.

## Results

Incorporating DPs in the basic ration to replace barley straw and the substitution of corn by CDs in the additional ration significantly influenced the final LW of the lambs in the experimental groups compared to the control group (Table-6). The ADG and ADI in the 0%, 50%, and 80% groups remain unchanged while those of the 100% group are significantly different (+46% and +7%, respectively). The FCR of the 100% group (5.08 g of DM/g) is significantly different from the other four groups.

**Table-6:** Fattening performances of Ouled Djellal lambs.

Parameters	L0	L50	L80	L100	SEM	p-value
Initial LW (kg)	32.3	31.7	32.1	32.4	0.43	0.95
Final LW (kg)	39.6^a^	40.1^ab^	40.5^ab^	43.1^b^	0.55	0.01
ADG (g)	130.35^a^	149.10^a^	150^a^	191.07^b^	5.47	0.001
ADI (g/animal/day)	900.9^a^	917.8^a^	922.3^a^	963.4^b^	12.56	0.001
FCR (g DM/g)	7.34^b^	6.49^b^	6.27^ab^	5.08^a^	0.25	0.01

In each line, the numbers followed by the same exponents do not differ significantly at p<0.05, SEM=Standard error of the mean, LW=Live weight, ADG=Average daily gain, ADI=Average daily intake, FCR=Feed conversion ratio, DM=Dry matter

**Figure-1:**
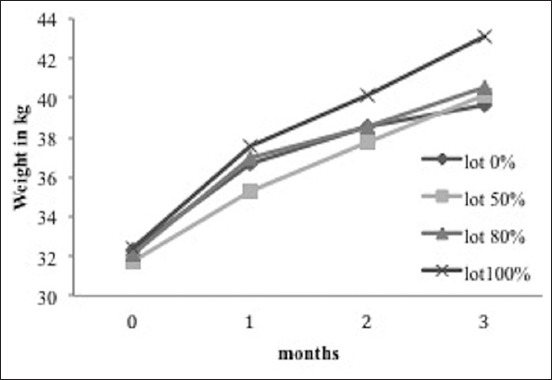
Change in live weight according to the substitution rates.

The 50% group displays the smallest change in LW for the experiment as a whole (Figure-1). The 0% and 80% groups show a similar variation in LW until the 60^th^ day, with a difference at the 90^th^ day in favor of the 80% group. For the 100% group, the change in LW is significantly higher than the other groups.

The measurements of the carcass characteristics are presented in Table-7. There is no significant difference in the HC and CC weight between the different groups. Drying in the cold room generated a difference in carcass weight of about 0.6 points. The type of diet significantly (p<0.05) influenced the carcass yield between the control group and the 100% group (44.66% vs. 48.77%, respectively). The yield of the 50% group was similar to that of the control group, whereas it was intermediate for the 80% group at 47.31%.

**Table-7:** Effects of incorporating DPs and cull dates on the characteristics of Ouled Djellal lamb carcasses.

Parameters	0	50	80	100	SEM	p-value
Hot carcass weight (kg)	20.13	20.26	20.46	22.93	0.57	0.28
Cold carcass weight (kg)	19.5	19.53	19.9	22.43	0.57	0.22
Carcass yield (%)	44.66^a^	45.35^a^	47.31^ab^	48.77^b^	0.63	0.01
Thickness of back fat (cm)	3.93^b^	3.46^a^	3.56^ab^	3.23^a^	0.09	0.02
Thickness of leg (cm)	30.66	27.13	30.23	30.9	0.9	0.47
Width of pelvis (G) (cm)	22.63	20.96	21.26	23.0	0.57	0.58
Width of chest (LAC) (cm)	19.16	19.83	19.30	20.56	0.62	0.89
Width of shoulders (M) (cm)	15.86	16.33	15.66	17.93	0.43	0.25
Length of carcass (K) (cm)	66.33	67.50	67.66^a^	68.00	0.95	0.95
Depth of chest (TH) (cm)	30.2	26.4	29.86	30.3	0.95	0.45
Length of leg (F) (cm)	33.96	37.00	35.00	36.83	0.69	0.37
Leg index (width/length)	0.66^b^	0.56^a^	0.60^ab^	0.62^ab^	0.015	0.03
Carcass index 1 (width/length)	0.29	0.29	0.28	0.30	0.008	0.91
Muscle index (thickness/length of leg)	0.90^b^	0.73^a^	0.86^ab^	0.84^ab^	0.026	0.01
Carcass index 2 (weight/length of carcass)	0.295	0.289	0.293	0.330	0.008	0.30
Conformation index (g/cm)	1.19^b^	1.02^a^	1.15^ab^	1.17^b^	0.026	0.02

DPs=Date pedicels, In each line, the numbers followed by the same exponents do not differ significantly at P<0.05, SEM=Standard error of the mean

A significant reduction (p=0.02) in back fat was recorded for the 50% and 100% groups compared to the control group. The 80% group displayed an intermediate thickness of back fat (3.56%) and remained lower than that of the control group (3.93%).

The thickness of the leg, width of the pelvis, shoulders and thorax, length of carcass, depth of chest, length of leg, carcass index 1, and carcass index 2 do not differ among the groups.

The leg and muscle indices differ between the 0% and 50% groups (p<0.05), while the 80% and 100% groups recorded similar intermediate values (approximately 0.61 and 0.85, respectively). The conformation index (sum of the muscle and carcass indices) is similar in the control group and the 100% group (approximately 1.18 g/cm), while the 50% group records the least expressive index (1.02 g/cm) and the 80% group displays an intermediate value (1.15 g/cm).

The by-products of the date palm and from the abattoir used have not only improved the lamb fattening performances but have also minimized the cost of feeding with a profit margin of +DZD (Algerian Dinars) 30.93/day and/animal (Table-8).

**Table-8:** Economic cost.

Raw materials	Price DZD/kg	% substitution			

		0		100	
	
		Qt (g)	Price	Qt (g)	Price
Control concentrate	45	900.89	40.54		
Experimental concentrate	42.63			963.39	41.07
Control basic R	20	500	10		
Experimental basic R	4.168			500	2.08
Price (DZD/d) ovine ration		50.54		43.15	
ADG (g)		130.35		191.07	
Price the consumer is willing to pay to have the same yield^1^		74.08^1^			
Profit margin (DZD/day/animal)^2^		30.93^2^			

N.B.=The food prices used are the local market prices,

1 Calculated by the rule of three: The farmer pays DZD 50.54 to have 130.35 g of grow, so for an grow of 191.07 g, he must pay DZD 74.08,

2 It is the difference between the price of the farmer’s ration and the experimental ration (100%) for an grow of 191.07 g (DZD 43.15 vs. 74.08), ADG=Average daily gain

### Cost price of DPs constituting the experimental basic ration

The DPs are date harvest and packaging waste. The annual tonnage can be estimated using the quantities of dates produced at national level (1,100,000 tonnes in 2017) [18] and multiplying this by 1.84% [4].

The quantity of pedicels will be 1,100,000×1.84%=20,240 tonnes for 2017, which are then sent as they are to landfill sites. Their recoveries will only cost transportation and labor costs. Accordingly:

- Transport costs will be DZD 5000 (truck with a capacity of 15 tonnes), given that the pedicel treatment plant is 20 km from the processing and/or packaging plant. The cost of transporting 1 kg of DPs to the treatment plant will be 0.33 DZD/kg/day.
- The labor tasked with loading, treating, and storing the pedicels is estimated at 8 workers paid 1200 DZD/day/worker, giving 8×1200 DZD/15,000 kg of DPs treated per day=0.64 DZD/kg/day.
- The cost of the treatment product is estimated at 3 DZD/kg, calculated as follows: 100 kg DPs treated using 5 kg urea, with one bag of 100 kg ammonitrate containing 46% urea costing 6000 DZD.

The direct variable cost is estimated at

DVC=3.97 DZD/kg DP treated

The indirect variable costs (IVC) are 5% of the DVC, i.e. 5×3.97/100=0.1985 DZD/kg

IVC=0.1985 DZD/kg DP treated

The total cost of 1 kg of DP treated using urea would be:

Total cost=3.97+0.1985=4.1685 DZD/kg DP treated

The cost price of 1 kg of LW of the 100% group fed with CDs, treated DPs, and RCE is highly advantageous at DZD 480.07 compared to DZD 824.93/kg for the 0% group, representing a 42% reduction (Table-9) [1]. The profit margin rises from a negative (DZD −174.93/kg LW) to a positive value (DZD +169.52/kg LW).

**Table-9:** Cost of producing 1 kg of live weight.

Costs	Control group	100% group	Difference
Feed costs (DZD/day)	50.54	43.15	−7.39
Production costs* (DZD/day)	107.53	91.8	−15.72
ADG (g)	130,35	191.07	−60.72
Production costs (DZD/kg)	824,93	480.07	−344.47
Sale price of 1 kg lamb LW**	650.00	650.00	-
Profit margin (DZD/kg LW)	−174.93	169.52	-

* Calculated by the rule of three; feed costs=47% of total production cost [1],

** Sale price of 1 kg lamb LW in the experimental farm during the period of Aïd El Kebîr, ADG=Average daily gain, LW=Live weight

## Discussion

The chemical composition of CDs varies from one author to another and is linked to the proportion of pits from the processing units (date paste) and dates rejected by the packaging units [19].

Incorporating CDs and the RCE significantly influenced the final weights of the Ouled Djellal lambs. Alhomidy *et al*. [20] mentioned that the use of CDs increases both LW and digestive efficiency among sheep. Bayati *et al*. [21] also claimed that the use of CDs as a source of energy enhances the efficiency of the use of nitrogen in the rumen. Javidan and Khezri [9] echoed this, indicating that the use of CDs is conducive to microbial protein synthesis in sheep.

Meradi *et al*. [7] and Al-Shantil *et al*. [22], nevertheless, detected non-significant differences in final LWs with 100% substitution of corn by CDs on local lambs. In Ardi goats given feed made from date pits and date palm seeds treated with urea, Al-Suwaiegh [8] found no significant difference in final LWs.

With regard to use of CDs in ruminant rations, Kholif and El-Nor [23] and Kholif *et al*. [24] have illustrated the need for protein supplements which are provided by the RCE in our additional ration. According to Ziolecka *et al*. [25], the RCE provides a suitable environment for the growth and development of lactobacillus, preventing the growth of pathogenic microorganisms (Clostridium, *Escherichia coli*, Salmonella, etc.), and increases the concentration of lactic acid. Şahin *et al*. [14] have shown that adding RCE to the rations fed to lambs and calves has enabled to improve fattening performances.

Treating DPs with urea has also made it possible to satisfy the maintenance needs and to contribute to an increase in the final weight of lambs, in particular, the 100% group as stressed by Nyarko-Badohu *et al*. [2].

Using waste dates from the processing and packaging process in the rations of feeder lambs increased the total integrated quantity per day for the 100% group (963.39 g), without for all that affecting the 0%, 50%, and 80% groups (900.89, 917.85, and 922.32 g, respectively). These results are consistent with those of Meradi *et al*. [7] and consolidated by those of Bayati Zadeh *et al*. [21]. The most expressive FCR is that of the 100% group, with 5.08 (p<0.05).

The ADG is highly significant (p<0.05) for the 100% batch (191.07 g/d) with +61 g/d compared to the control group. Chehma and Longo [26] found that incorporating 75% CDs results in an ADG of 150 g and concludes that the CDs have the same properties as an energy shot for sheep fattening.

The carcass yield was most expressive for the 80% and 100% groups, at 47.31% and 48.77%, respectively. This improvement is also observed by Farhan *et al*. [27], for a diet based on date pits. The variation in the thickness of back fat was less expressive for the 100% group (3.23 cm) and was due to the combination of the nitrogen provided by the pedicel treatment and the rate of CDs (60%) which synthesized microbial protein in the rumen, giving rise to a final weight of 43.10 kg and reducing the lipogenesis of the sugars provided by the CDs. The conformation rate was not significant between the 0% and 100% groups, echoing the observations of Sañudo *et al*. [28] who noted that diet has little impact on this index.

## Conclusion

The use of by-products of the date palm (CDs and pedicels) combined with RCE in animal feed with a view to fattening sheep, in particular in oasis zones, represents an alternative in enhancing growth performances and carcass characteristics and offers a relatively cheap prospect for the availability of red meat for populations with low purchasing power.

## Authors’ Contributions

AB prepared the ground conditions and collected the data, RA revised the manuscript, EM carried out and drafted the economic analysis, YA performed the analysis of the data, FA designed the study and drafted, and HSA revised the manuscript. All authors have read and approved the final manuscript.
